# The Synaptic Vesicle Protein 2A Interacts With Key Pathogenic Factors in Alzheimer’s Disease: Implications for Treatment

**DOI:** 10.3389/fcell.2021.609908

**Published:** 2021-07-01

**Authors:** Yanyan Kong, Lin Huang, Weihao Li, Xuanting Liu, Yinping Zhou, Cuiping Liu, Shibo Zhang, Fang Xie, Zhengwei Zhang, Donglang Jiang, Weiyan Zhou, Ruiqing Ni, Chencheng Zhang, Bomin Sun, Jiao Wang, Yihui Guan

**Affiliations:** ^1^PET Center, Huashan Hospital, Fudan University, Shanghai, China; ^2^Laboratory of Molecular Neural Biology, School of Life Sciences, Shanghai University, Shanghai, China; ^3^Department of Neurosurgery, Ruijin Hospital, Shanghai Jiao Tong University School of Medicine, Shanghai, China; ^4^Institute for Biomedical Engineering, ETH Zurich, University of Zurich, Zurich, Switzerland

**Keywords:** Alzheimer’s disease, neurodegeneration, synaptic vesicle protein 2A, Tau, Aβ, PI3K signaling pathway

## Abstract

Alzheimer’s disease (AD), a serious neurodegenerative disease, is pathologically characterized by synaptic loss and dysfunction. Synaptic vesicle protein 2A (SV2A) is an indispensable vesicular protein specifically expressed in synapses and can be used as a biomarker for synaptic density. We found that the expression of SV2A was down-regulated in the hippocampus of AD patients, yet the relation of SV2A to other hallmarks of AD pathology such as amyloid precursor protein (APP), β-amyloid (Aβ), and Tau protein is not thoroughly clear. In addition, SV2A colocalized with APP and was down-regulated at Aβ deposition. Moreover, we found that SV2A deficiency leads to a simultaneous increase in Aβ and Tau hyperphosphorylation, while SV2A overexpression was associated with downregulation of β-site APP cleaving enzyme 1 and apolipoprotein E genes. In addition, evidence gained in the study points to the phosphatidylinositol 3-kinase signaling pathway as a possible mediator in SV2A regulation influencing the incidence and development of AD. With limited effective diagnostic methods for AD, a close interplay between SV2A and AD-related proteins demonstrated in our study may provide novel and innovative diagnostic and therapeutic opportunities.

## Introduction

Alzheimer’s disease (AD) is the most common progressive neurodegenerative disease associated with aging (Heneka et al., 2015; Turab Naqvi et al., 2020). Typical pathological features of AD include senile plaques and neurofibrillary tangles (Ulrich et al., 2014; Wang et al., 2016). The primary component of senile plaques is β-amyloid (Aβ), which is secreted as an enzymatic digestion product of amyloid β-protein precursor (APP) by hydrolase (Myllykangas et al., 2002; Reed-Geaghan et al., 2009; Magnoni et al., 2012; Kummer et al., 2015), while neurofibrillary tangles are composed of hyperphosphorylated tau proteins (Noble et al., 2013; Rodriguez-Martin et al., 2013; Pooler et al., 2014; Frost et al., 2015; Mcinnes et al., 2018; Turab Naqvi et al., 2020). Aβ deposition leads to senile plaques and neurofibrillary tangles, which cause damage and loss of brain neurons, compromise neurological function, and ultimately result in AD dementia (Myllykangas et al., 2002; Kramer et al., 2004; Mcconlogue et al., 2007; Silvestre et al., 2017). However, the exact regulatory mechanisms of AD are complex and remain to be fully elucidated. Understanding the pathogenic mechanisms of Aβ and tau in the AD brain is particularly important for AD prevention and treatment (Buckley and Kelly, 1985; Ma et al., 2018; Mcinnes et al., 2018).

Synaptic loss and synaptic dysfunction are well-established major mechanisms of the pathological basis of mild cognitive impairment (MCI) in early AD and the structural basis of AD dementia (Hatanpaa et al., 1999; Kelley and Petersen, 2007; Chen et al., 2018; Lauterborn et al., 2019). However, the specific molecular mechanisms leading to compromised synaptic function remains unclear. Synaptic vesicle glycoprotein 2A (SV2A), an essential vesicle membrane protein ubiquitously expressed in synapses, could serve as a suitable biomarker for synaptic density (Madeo et al., 2014; Nicolas et al., 2016; Stockburger et al., 2016; Tokudome et al., 2016; Chen et al., 2018; Rokka et al., 2019). SV2A is also involved in synaptic vesicle transport, exocytosis, neurotransmitter release, and regulates gene and protein expression (Cohen et al., 2011; Mendoza-Torreblanca et al., 2013). Clinical studies have shown that SV2A dysfunction is involved in the pathogenesis of AD (Mendoza-Torreblanca et al., 2013; Löscher et al., 2016; Metaxas et al., 2019). For example, positron emission studies using the SV2A radiotracer ^11^C-UCB-J and ^18^F-UCB-H have shown that AD patients had significantly less SV2A binding in the hippocampus compared to non-AD subjects (Chen et al., 2018; Bastin et al., 2020). Therefore, targeting SV2A may provide a novel strategy for early diagnosis and treatment of AD (Kramer et al., 2004; Tokudome et al., 2016; Onwordi et al., 2020).

In this study, we found that overexpressing and silencing SV2A induced changes in the expression level of Aβ both *in vitro* and *in vivo*. Our results indicate that upregulation of SV2A decreases the relative expression level of AD-related genes. We found that Aβ expressions were significantly increased in certain brain regions of SV2A-knockout (KO) mice by positron emission tomography (PET) imaging techniques. Moreover, we found that SV2A regulation of the occurrence and development of AD appeared to be mediated by the phosphatidylinositol 3-kinase (PI3K) signaling pathway. Our research provides evidence that SV2A is an important regulator of AD and lays the foundation for further research on neurological diseases.

## Materials and Methods

### Mouse Model

The Sv2a gene (NCBI reference sequence: NM_022030.3) is located on mouse chromosome 3. Thirteen exons have been identified, with the ATG start codon in exon 2 and the TGA stop codon in exon 13. Exon 3 will be selected as a conditional KO (cKO) region. Deletion of this region should result in the loss of function of the mouse Sv2a gene. In the targeting vector, the “SA-2A-dTomato-polyA” cassette will be cloned into intron 3 in the reverse orientation. The cKO region and the reverse “SA-2A-dTomato-polyA” cassette will be flanked with loxP and lox2272 sites. To engineer the targeting vector, homology arms and the cKO region will be generated by PCR using BAC clones RP24-293K7 and RP23-16I15 from the C57BL/6 library as a template. In the targeting vector, the Neo cassette will be flanked by SDA (self-deletion anchor) sites. DTA will be used for negative selection. The constitutive KO allele will be obtained after Cre-mediated recombination.

### PET

Positron emission tomography experiments were performed using a Siemens Inveon PET/CT system (Siemens Medical Solutions, Knoxville, United States) and conducted by the Huashan Hospital affiliated with Fudan University of China. PET/CT imaging of the mice brains was analyzed using PMOD software (version 3.4, PMOD Technologies Ltd., Zurich, Switzerland). Static PET/CT imaging was obtained for 15 min at 50 min after the intravenous administration of [^18^F]-AV45 (∼0.37 MBq/g body weight). PET/CT images were reconstructed using the ordered subsets expectation maximization 3D algorithm, and the data were reviewed and processed using the IRW and analyzed with PMOD software (Ottoy et al., 2019). All experiments were carried out in compliance with national laws for the conduct of animal experimentation and were approved by the Animal Ethics Committee of Fudan University.

### ELISA

Ninety-six-well plates (R&D Systems, United States) were washed three times with wash buffer immediately prior to use. All reagents, working standards, and samples were prepared as directed in the previous sections. One hundred microliters of standard, control, and samples was added and incubated for 2 h at 4°C. Each well was aspirated and washed, then added with two hundred microliters of cold human Aβ (Aβ1–42) conjugates to each well, covered with a new adhesive strip, and incubated for 2 h at 4°C. Two hundred microliters of substrate solution was added to each well and incubated for 30 min at room temperature (RT) on the benchtop. Fifty microliters of stop solution was added to each well. The optical density of each well was determined within 30 min, using a microplate reader set to 450 nm.

### Database Analysis

The datasets used were from the Aging, Dementia, and Traumatic Brain Injury Study^1^ (Miller et al., 2017) and included 304 RNA-seq samples collected from the hippocampus of 108 elderly donors with AD. We compared the amount of SV2A expression according to their normalized fragments per kilobase of transcript per million fragments mapped (FPKM) values.

### Immunohistochemical Staining

Frozen sectioning was used to slice brain samples from AD patients and non-AD patients; samples were donated by the Department of Anatomy, Histology, and Embryology (School of Basic Medicine, Fudan University, Shanghai, China). Sections (20 μm) were rinsed three times with PBS and then permeabilized with 0.1% Triton X-100 in PBS, blocked with 5% BSA in PBS at RT for 30 min, and incubated overnight at RT with both rabbit SV2A antibody (1 μg/ml, Abcam) and mouse APP antibody (1 μg/ml, Covance). Subsequently, sections were washed three times in PBS, followed by a 2-h incubation with goat anti-rabbit IgG (594; 3 μg/ml, Abcam) and goat anti-mouse IgG (488; 3 μg/ml, Abcam). Sections were again washed three times with PBS. Fluorescence intensity was detected by using a Zeiss LSM710 fluorescence microscope (Liu et al., 2018).

### Genotyping by PCR

Genomic DNA extracted from the tail was amplified by PCR using primers for the SV2A gene (forward, 5′-GAGGCTGTCTACACTGAGGTCTACTG-3′) and (reverse, 5′-TGCGAGGCCAGAGGCCACTTGTGTAGC-3′). The first cycle used 94°C for 3 min, followed by 33 cycles at 94°C for 30 s, 62°C for 35 s, and 72°C for 20 s. To identify mice with the targeted allele, the following primers were used to produce a 293-bp amplicon in SV2A mice and a 183-bp amplicon in WT mice: forward 5′-GAGGCTGTCTACACTGAGGTCTACTG-3′ and reverse 5′-CATAGCTGTCCCTCTTCTCTTATGGAG-3′.

### Cell Culture

The APPswe293T cell line, a human renal epithelial cell line expressing the SV40 T antigen, was kindly provided by Prof. F. Huang (Shanghai Advanced Research Institute, Chinese Academy of Sciences, Shanghai, China). It is a stable cell line that can continuously express APP and secrete Aβ into the medium. Cells were maintained in Dulbecco’s modified Eagle Medium (Invitrogen, United States) containing 10% fetal bovine serum (Invitrogen, United States) and 1% penicillin/streptomycin (Invitrogen, United States), 0.1% G418 (Invitrogen, United States). The cells were cultured in a humidified incubator with 5% CO_2_ at 37°C. When the cells reached 80–90% confluence, they were detached with 0.25% trypsin (Gibco, Canada), seeded onto appropriate plates with fresh medium, and incubated overnight.

### Infection

APPswe293T cells were seeded onto 12-well plates at a density of 2 × 10^5^ cells per well and cultured overnight at 37°C in an atmosphere of 5% CO_2_. The following day, cells were infected with the SV2A-overexpressing virus (Lenti-CMV-SV2a-RFP), overexpressing virus control (Lenti-EGFP-Puro), SV2A-silencing virus (Lenti-CMV-SV2a-shRNA-Puro), or a silencing virus control (Lenti-shRNA-mCherry), according to the manufacturer’s protocol.

### Immunofluorescence

APPswe293T cells were cultured in 12-well plates and infected as previously described; bright-field images were acquired using a Nikon microscope. Cells were then rinsed with PBS, fixed in 4% paraformaldehyde in PBS at RT for 10 min, and permeabilized with 0.1% Triton X-100 in PBS for 10 min. Subsequently, the cells were blocked in 2% bovine serum albumin in PBS at RT for 1 h, followed by overnight incubation with rabbit anti-BACE1 polyclonal primary antibody (2 μg/ml, Abcam, United States) and mouse anti-APP monoclonal primary antibody (2 μg/ml, Cell Signaling Technology) at 4°C. Cells were then washed three times with PBS and incubated with three antibodies: donkey anti-mouse IgG secondary antibody Alexa 488 (1 μg/ml, Abcam, United States), donkey anti-rabbit IgG secondary antibody Alexa 594 (1 μg/ml, Abcam, United States), and donkey anti-goat mouse secondary antibody Alexa 647 (1 μg/ml, Abcam, United States). The incubation sustained at RT for 2 h. Then DAPI was used following incubation at RT for 5 min. Finally, the cells were washed three times with PBS. Fluorescence intensity was detected using a Zeiss LSM710 fluorescence microscope.

### Total RNA Extraction, cDNA Synthesis, and Real-Time Quantitative PCR

APPswe293T cells were cultured in 12-well plates and infected as described above. Total RNA was extracted using a total RNA extraction kit (Promega, United States), 48 h post infection, according to the manufacturer’s protocol. The concentration of RNA was determined by measuring the absorbance at 260 nm, and 2 μg RNA was used for cDNA synthesis using an RT Master Mix (TaKaRa, Japan). Quantitative PCR (qPCR) amplification was performed at least three times using a mixture of SYBR Green qPCR super mix (YEASEN, China), cDNA samples, and designated primers (Table 1). The relative gene expression levels were calculated by comparing the CT value of the gene of interest with that of Gapdh, the internal control.

**TABLE 1:** List of primers used for qPCR.

**Gene name**	**Primer sequence (5′–3′)**
*GAPDH*	Upstream: GGAGCGAGATCCCTCCAAAAT
	Downstream: GGCTGTTGTCATACTTCTCATGG
*APOE*	Upstream: GTTGCTGGTCACATTCCTGG
	Downstream: GCAGGTAATCCCAAAAGCGAC
*APP*	Upstream: CTGGAGGGTATGGGGTTCC
	Downstream: TTGTCTTGCAGGACGTAGGTC
*TAU*	Upstream: GCTGTGGCAGGAACGAGAA
	Downstream: AGTTTGAAGGCTTGATGTCACG
*BACE1*	Upstream: TCTGTCGGAGGGAGCATGAT
	Downstream: GCAAACGAAGGTTGGTGGT
*SV2A*	Upstream: GCACAACGACGGAAAGAACG
	Downstream: CATGCCTTTGTTGGAGTCGG

### Western Blotting

The total protein in the APPswe293T cells was extracted using a cell lysis buffer (Beyotime, China), according to the manufacturer’s protocol. The infected cells were in 12-well plates and the protein was extracted from the APPswe293T cells. After treatment, cells were washed twice with ice-cold PBS, and the total protein was extracted using cell lysis buffer (Beyotime, China), according to the manufacturer’s protocol. Protein samples were separated by sodium dodecyl sulfate–polyacrylamide gel electrophoresis and electroblotted onto nitrocellulose membranes. The membranes were blocked with 5% bovine serum albumin in PBS at RT for 1 h and then incubated with the following primary antibodies at 4°C overnight: mouse anti-APP (1 μg/ml, Cell Signaling Technology), rabbit anti-tau (1 μg/ml, Abways, China), rabbit anti-BACE1 (1:1,000, Abcam, United States), rabbit anti-APOE (1 μg/ml, ABclonal, China), mouse anti-PI3K (0.25 μg/ml, Proteintech, China), rabbit anti-ERK (1 μg/ml, Proteintech, China), and rabbit anti-SRK (1 μg/ml, Proteintech, China). The following day, the membranes were incubated with a mouse anti-GAPDH (1 μg/ml, Abcam, United States) at RT for 1 h, followed by an infrared dye 700-conjugated goat anti-mouse IgG (0.1 μg/ml, Zemed, United States) and an infrared dye 800-conjugated goat anti-rabbit IgG (0.1 μg/ml, Zemed, United States) at RT for another hour. Visualization and quantification were carried out using the LI-COR Odyssey scanner and associated software (LI-COR Biosciences). The relative protein expression level was normalized to the Gapdh value from the same lane. Data were obtained from four immunoblots.

### PI3K Inhibition Experiment

The cultured APPswe293T cells were divided into four groups: control, SV2A, shControl, and shSV2A groups, and the corresponding four groups were treated with PI3K inhibitor LY294002 (50 μm). After 48 h of infection, cells were treated or not with 50 μm LY294002 for 4 h, protein was extracted, and the expression of PI3K signaling-related molecules was detected by western blotting.

### Statistical Analysis

All data were analyzed using the GraphPad Prism software and were presented as the mean ± SEM. The mRNA and protein expression levels of cells were analyzed using a *t*-test. The microscope images were analyzed using the Image Pro Plus software. Significance was set to *p* < 0.05.

## Results

### SV2A Is Down-Regulated in the Brain of AD Patients and Colocalized With APP

**FIGURE 1:**
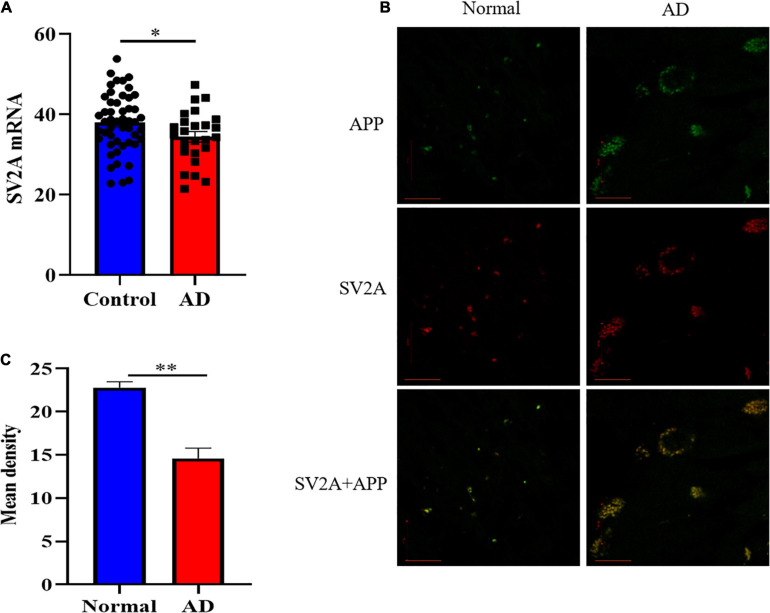
SV2A expression decreased and its pattern changed in AD patients. **(A)** Comparation of relative SV2A mRNA expression levels in the hippocampus of non-AD patients and AD patients. The expression level was normalized to the mean expression level of non-AD patients. Data come from the Allen Brain database. For non-AD patients and AD patients, *n* = 50 and 25, respectively. **(B)** Immunofluorescence observation of APP (green) and SV2A protein (red) in the hippocampus of non-AD patients and AD patients reveals a change in the morphology of SV2A protein and a colocalization of APP and SV2A protein in the hippocampus of AD patients. Scale bar, 20 μm. **(C)** Quantitation of the mean density of SV2A staining in normal and AD brain sections. Data are expressed as mean ± SEM. One-way analysis of variance (ANOVA). ^∗^*p* < 0.05. ^∗∗^*p* < 0.01.

In order to investigate the molecular mechanism with which SV2A may be involved in the nervous system, we first examined and compared the mRNA levels of SV2A in the hippocampus of 108 AD patients and non-AD subject in the Allen Brain database (Miller et al., 2017). Statistical analysis shows that the mRNA levels of SV2A were significantly decreased in AD patients as compared with the non-AD project (Figure 1A). This result corroborates *in vivo* findings of reduced SV2A in the hippocampus of AD and MCI patients from SV2A PET imaging studies, and this suggests that SV2A plays an important role in the molecular mechanism of AD (Chen et al., 2018; Bastin et al., 2020). *In vivo* results showed significant changes of SV2A in AD patients’ brain compared with non-AD patients, and it was consistent with the location of Aβ deposition, suggesting that SV2A related to the pathological changes of AD (Figures 1B,C). For further analysis, we produced an SV2A-KO mice model deletion of exons 3, which resulted in the functional loss of the *SV2A* gene (Supplementary Figure 1D). PCR genotyping was used to identify homozygous KO mice from their wild-type (WT) and heterozygous littermates (Supplementary Figure 1C).

### Abrogation of SV2A Promotes Aβ Production

**FIGURE 2:**
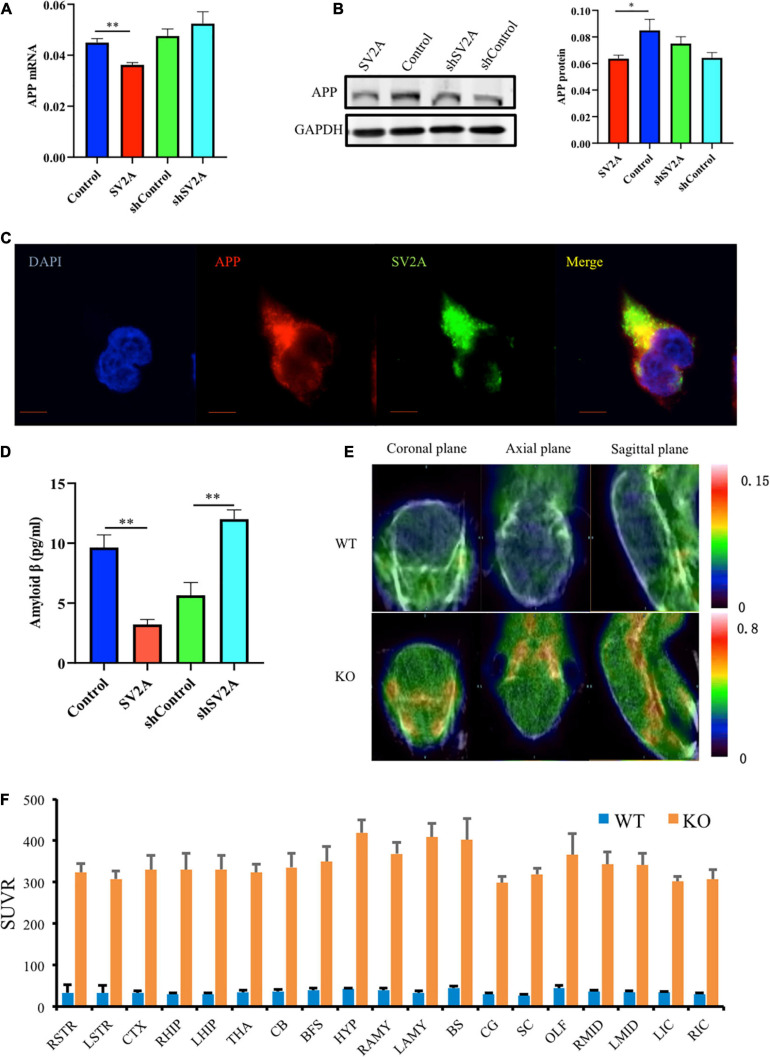
SV2A regulates the expression level of APP. **(A)** qPCR detection of APP mRNA expression level in APPswe293T cells from the following four groups: control, cells infected with the SV2A-overexpressing virus (SV2A), shControl, and cells infected with the SV2A-silencing virus (shSV2A). Gapdh was used as the internal control. **(B)** Western blotting detection of APP expression levels in APPswe293T cells from the following four groups: control, cells infected with the SV2A-overexpressing virus (SV2A), shControl, and cells infected with the SV2A-silencing virus (shSV2A). The gray density was normalized to the mean gray density of Gapdh. **(C)** Immunofluorescence observation of the APP (red) and SV2A (green) in APPswe293T cells. The nuclei were counterstained with DAPI (blue). Scale bar, 40 μm. **(D)** Statistical analysis of ELISA detection of the expression of Aβ secreted from APPswe293T cells from the following four groups: control, cells infected with the SV2A-overexpressing virus (SV2A), shControl, and cells infected with the SV2A-silencing virus (shSV2A). **(E)** ^18^F-AV45 PET was used to trace Aβ in WT and SV2A-KO mice. **(F)** The ^18^F-AV45 SUVR of different brain regions in WT and SV2A-KO mice using PMOD analysis. For each group of APPswe293T cells, *n* = 3. Data are expressed as mean ± SEM. One-way analysis of variance (ANOVA). ^∗^*p* < 0.05. ^∗∗^*p* < 0.01.

Senile plaques, a by-product of APP hydrolysis, is a key component of the neurodegeneration pathogenesis observed in AD (Espeseth et al., 2005; Mastromoro et al., 2019). To further explore the relationship of SV2A and Aβ, APPswe293T cells were cultured *in vitro*. After infection with the SV2A virus, RNA and protein were extracted from cells and analyzed by quantitative real-time polymerase chain reaction (PCR) and western blotting. In addition, the infection effect of the virus has been verified (Supplementary Figure 1A). Overexpression of SV2A decreased the RNA level of APP (Figure 2A). The agarose gel electrophoresis result of the product was consistent with qPCR analysis results (Supplementary Figure 1B). Conversely, the expression of APP proteins was up-regulated in the SV2A-silencing virus group compared to the control group (Figure 2B), indicating that SV2A may regulate AD pathogenesis through AD-associated proteins. In addition, APP and SV2A are colocalized in APPswe293T cells (Figure 2C).

By using ELISA, the expression of Aβ secreted in the medium was assessed, 48 h post infection, and it was found that SV2A had a negative effect on Aβ expression. Overexpression of SV2A decreased the expression level of Aβ, and the downregulation of SV2A up-regulated the expression of Aβ (Figure 2D). Using micro PET imaging, we also compared the expression of Aβ in SV2A-KO mice and WT mice, showing that Aβ was over-accumulated in the brains of SV2A-KO mice, which further confirmed previous results (Figure 2E). The ^18^F-AV45 SUVR was analyzed by PMOD of different brain regions in WT and SV2A-KO mice (Figure 2F).

### SV2A Regulates the Expression Level of BACE1 and APOE

**FIGURE 3:**
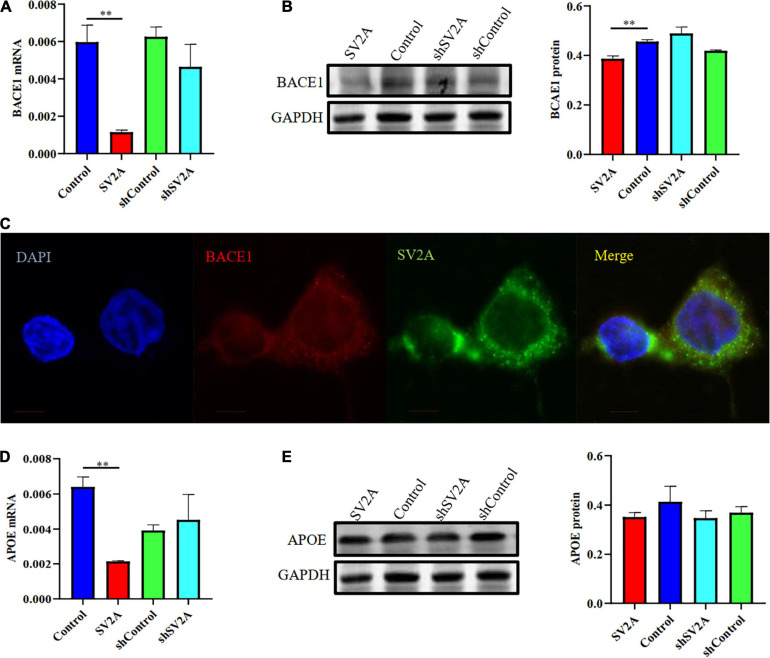
SV2A regulates the expression level of BACE1 and APOE. **(A)** qPCR detection of BACE1 mRNA expression level in APPswe293T cells from the following four groups: control, cells infected with the SV2A-overexpressing virus (SV2A), shControl, and cells infected with the SV2A-silencing virus (shSV2A). Gapdh was used as the internal control. **(B)** Western blotting detection of BACE1 expression levels in APPswe293T cells from the following four groups: control, cells infected with the SV2A-overexpressing virus (SV2A), shControl, and cells infected with the SV2A-silencing virus (shSV2A). The gray density was normalized to the mean gray density of Gapdh. **(C)** Immunofluorescence observation of the BACE1 (red) and SV2A (green) in APPswe293T cells. The nuclei were counterstained with DAPI (blue). Scale bar, 40 μm. **(D)** qPCR detection of the APOE mRNA expression level in APPswe293T cells from the following four groups: control, cells infected with the SV2A-overexpressing virus (SV2A), shControl, and cells infected with the SV2A-silencing virus (shSV2A). Gapdh was used as the internal control. **(E)** Western blotting detection of APOE expression levels in APPswe293T cells from the following four groups: control, cells infected with the SV2A-overexpressing virus (SV2A), shControl, and cells infected with the SV2A-silencing virus (shSV2A). The gray density was normalized to the mean gray density of Gapdh. For each group of APPswe293T cells, *n* = 3. Data are expressed as mean ± SEM. One-way analysis of variance (ANOVA). **p < 0.01.

We investigated the β-site APP cleaving enzyme 1 (BACE1) and the apolipoprotein E genes (APOE), two other genes associated with AD, for their relationship with SV2A. They were selected because BACE1 is the first enzyme involved in APP splicing (Bettens et al., 2009; Ridler, 2018), and APOE is a well-known genetic risk factor for late-onset AD (LOAD) (Schreiber et al., 2017; Prendecki et al., 2019; Weintraub et al., 2019). We found that cells infected with the SV2A-overexpressing virus showed a significant decrease in BACE1 expression as observed at the mRNA level (Figure 3A). The agarose gel electrophoresis result of the product was consistent with qPCR analysis results (Supplementary Figure 1B). Western blotting analysis showed similar results (Figure 3B). In addition, immunofluorescence observations of BACE1 and SV2A in APPswe293T cells revealed that they are colocalized (Figure 3C). Consistent with the BACE1 results, cells after transfection of a SV2A-overexpressing virus showed a lower level of APOE expression (Figure 3D). The agarose gel electrophoresis result of the product was consistent with qPCR analysis results (Supplementary Figure 1B). There was no significant difference between the samples observed by western blotting (Figure 3E). Overall, these results indicated that SV2A impacts Aβ production by interacting with the AD-associated genes (Figures 2, 3).

### SV2A Deficiency Promotes Tau Hyperphosphorylation

**FIGURE 4:**
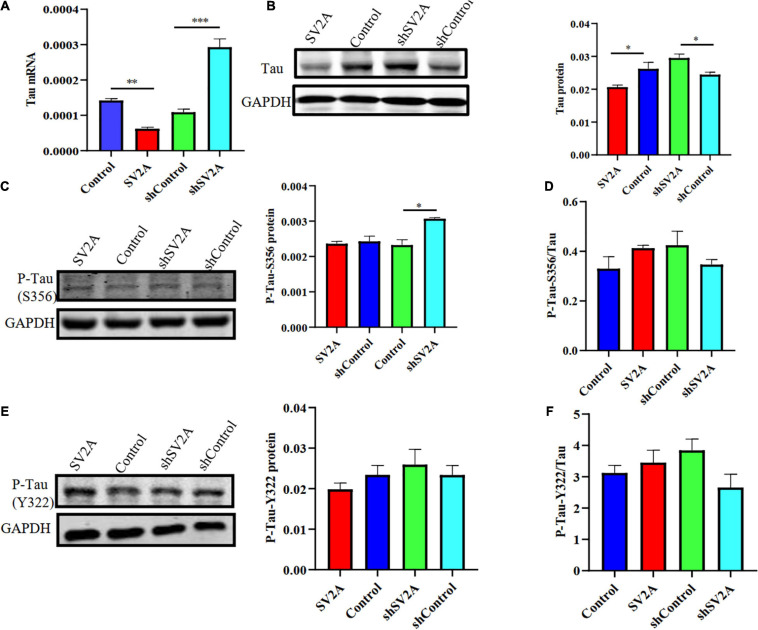
SV2A deficiency promotes tau hyperphosphorylation. **(A)** qPCR detection of the tau mRNA expression level in APPswe293T cells from the following four groups: control, cells infected with the SV2A-overexpressing virus (SV2A), shControl, and cells infected with the SV2A-silencing virus (shSV2A). Gapdh was used as the internal control. **(B)** Western blotting detection of tau expression levels in APPswe293T cells from the following four groups: control, cells infected with the SV2A-overexpressing virus (SV2A), shControl, and cells infected with the SV2A-silencing virus (shSV2A). The gray density was normalized to the mean gray density of Gapdh. **(C)** Western blotting detection of P-Tau-S356 protein (phosphorylated tau protein mutated at serine 356) expression levels in APPswe293T cells from the following four groups: control, cells infected with the SV2A-overexpressing virus (SV2A), shControl, and cells infected with the SV2A-silencing virus (shSV2A). The gray density was normalized to the mean gray density of Gapdh. **(D)** Ratio of P-Tau-S356 and tau protein expression levels in APPswe293T cells from the following four groups: control, cells infected with the SV2A-overexpressing virus (SV2A), shControl, and cells infected with the SV2A-silencing virus (shSV2A). The gray density was normalized to the mean gray density of Gapdh. **(E)** Western blotting detection of the P-Tau-Y322 protein (phosphorylated tau protein mutated at tyrosine 322) expression levels in APPswe293T cells from the following four groups: control, cells infected with the SV2A-overexpressing virus (SV2A), shControl, and cells infected with the SV2A-silencing virus (shSV2A). The gray density was normalized to the mean gray density of Gapdh. **(F)** Ratio of P-Tau-Y322 and tau protein expression levels in APPswe293T cells from the following four groups: control, cells infected with the SV2A-overexpressing virus (SV2A), shControl, and cells infected with the SV2A-silencing virus (shSV2A). The gray density was normalized to the mean gray density of Gapdh. For each group of APPswe293T cells, *n* = 3. Data are expressed as mean ± SEM. One-way analysis of variance (ANOVA). *P < 0.05, **P < 0.01, ***P < 0.001.

Another typical pathological feature of AD is the formation of neurofibrillary tangles, composed of hyperphosphorylated tau proteins (Cho et al., 2019). Thus, we examined whether SV2A regulates the expression level of hyperphosphorylated tau proteins by using APPswe293T cells infected with the SV2A-overexpressing and SV2A-silencing virus. Results revealed that total tau protein expression was down-regulated in SV2A-overexpressing cells as observed at the mRNA level, while total tau protein expression was not significantly different in SV2A-silencing cells (Figure 4A). The agarose gel electrophoresis result of the product was consistent with qPCR analysis results (Supplementary Figure 1B). Western blotting results also revealed a downregulation of the total tau protein expression in the SV2A-overexpressing cells, alongside an upregulation of total tau protein expression in SV2A-silencing cells (Figure 4B). We then assessed the expression level of the hyperphosphorylated tau protein mutated at serine 356 (P-Tau-S356) and the hyperphosphorylated tau protein mutated at tyrosine 322 (P-Tau-Y322) by western blotting (Figures 4C–F). Results demonstrated an upregulation of P-Tau-S356 expression level in SV2A-silencing cells. Taken together, these results indicate that SV2A is involved in AD by regulating the expression of hyperphosphorylated tau proteins (Figure 4).

### SV2A Regulated AD Through the PI3K Signaling Pathway but Not ERK or SRC

**FIGURE 5:**
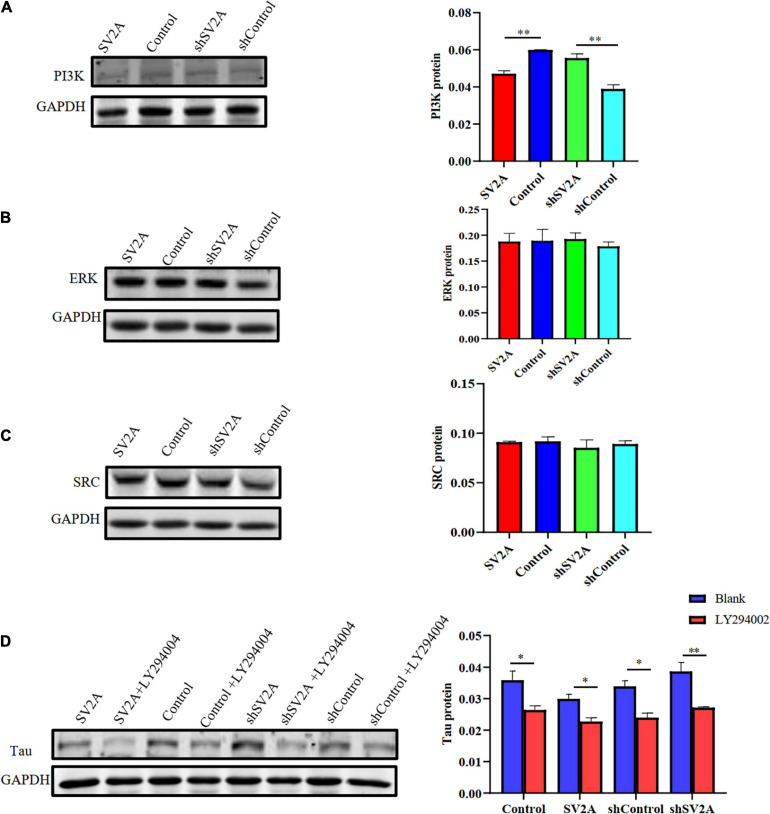
Overexpressing of SV2A inhibits the PI3K signaling pathway. **(A)** Western blotting detection of PI3K protein expression levels in APPswe293T cells from the following four groups: control, cells infected with the SV2A-overexpressing virus (SV2A), shControl, and cells infected with the SV2A-silencing virus (shSV2A). The gray density was normalized to the mean gray density of Gapdh. **(B)** Western blotting detection of ERK protein expression levels in APPswe293T cells from the following four groups: control, cells infected with the SV2A-overexpressing virus (SV2A), shControl, and cells infected with the SV2A-silencing virus (shSV2A). The gray density was normalized to the mean gray density of Gapdh. **(C)** Western blotting detection of SRC protein expression levels in APPswe293T cells from the following four groups: control, cells infected with the SV2A-overexpressing virus (SV2A), shControl, and cells infected with the SV2A-silencing virus (shSV2A). The gray density was normalized to the mean gray density of Gapdh. **(D)** Comparison of western blotting detection of tau protein expression levels in APPswe293T cells and APPswe293T cells with LY294002 (PI3K signaling pathway inhibitor) from the following four groups: control, cells infected with the SV2A-overexpressing virus (SV2A), shControl, and cells infected with the SV2A-silencing virus (shSV2A). The gray density was normalized to the mean gray density of Gapdh. For each group of APPswe293T cells, *n* = 3. Data are expressed as mean ± SEM. One-way analysis of variance (ANOVA). ^∗^*p* < 0.05. ^∗∗^*p* < 0.01.

We next assessed the dynamics of SV2A-related signaling pathways. Results revealed that compared with that of the control group, the expression of PI3K was significantly down-regulated at the protein level after infection with the SV2A-overexpressing virus and was significantly up-regulated after infection with the SV2A-silencing virus (Figure 5A). In contrast, the ERK and SRC signaling pathways showed no significant changes (Figures 5B,C). To further validate these observations, APPswe293T cells were treated with a PI3K-specific inhibitor, LY294002 (Wang et al., 2017), and showed that the expression of PI3K was down-regulated in all groups. We then compared the tau expression levels between SV2A-overexpressing cells and SV2A-silencing cells treated with LY294002 and found no significant difference (Figure 5D). In light of the above results, it can be speculated that SV2A influences AD via the PI3K signaling pathway.

## Discussion

**FIGURE 6:**
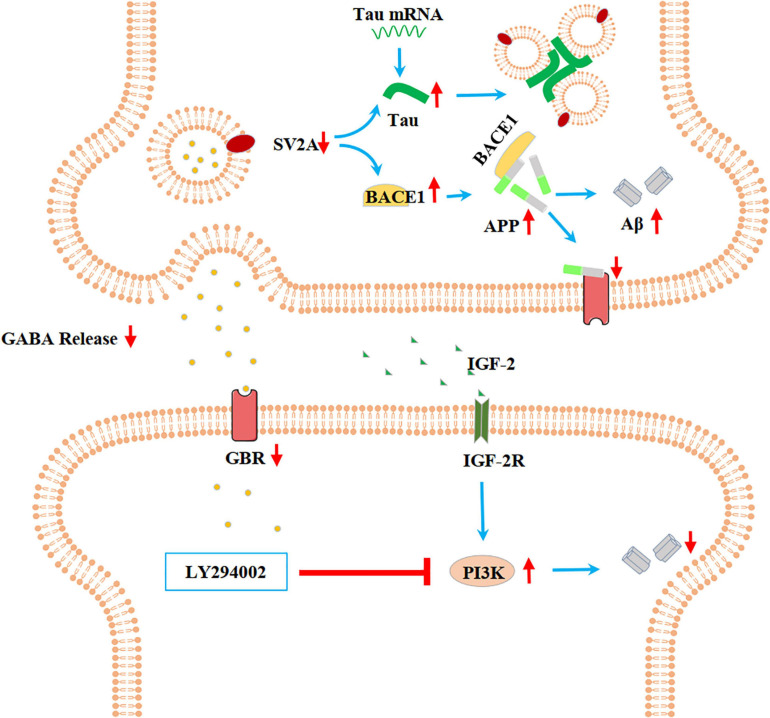
Schematic diagram showing that the deficiency of SV2A up-regulates Tau expression to reduce the inhibition of the impairment of synaptic vesicle information transmission, up-regulates BACE1 expression, and inhibits GBR-stabilizing APP on the cell surface, thus promoting the proteolysis of APP to Aβ. SV2A also regulates the PI3K pathway. SV2A regulates AD-related proteins via the above three ways.

In the present paper, we found SV2A to be colocalized with APP and down-regulated in the hippocampus of AD patients (Figure 1). Moreover, we showed that abrogation of SV2A promotes Aβ production (Figure 2) and that upregulation of SV2A down-regulates the AD risk factors BACE1 and APOE (Figure 3). Furthermore, SV2A deficiency promotes tau hyperphosphorylation (Figure 4). Finally, SV2A regulation of the pathogenesis and development of AD appear to be mediated by the PI3K signaling pathway (Figure 5). A schematic diagram showing that the deficiency of SV2A up-regulates tau expression to reduce the inhibition of the impairment of synaptic vesicle information transmission up-regulates BACE1 expression and inhibits GBR-stabilizing APP on the cell surface, thus promoting the proteolysis of APP to Aβ. SV2A also regulates the PI3K pathway (Figure 6). This study provides guidelines and information regarding the influence of the SV2A mechanism on the regulation of AD and possible future research of neurological diseases.

It has been shown that people with AD had significantly less SV2A binding in the hippocampus compared to cognitively normal participants according to SV2A PET using ^11^C-UCB-J, ^18^F-UCB-H, and ^18^F-SDM-8 (Chen et al., 2018; Bastin et al., 2020). Here, we found a significant decrease of SV2A expression at the mRNA level in the hippocampus of AD patients compared with non-AD subjects as per the Allen Brain database analysis (Figure 1), indicating that changes in SV2A expression influence synaptic function in AD and suggesting that SV2A may serve as a key regulator to AD-related proteins, such as Aβ and APP, two hallmarks of AD (Muller et al., 2017). Our study detected an increase in the expression levels of APP in APPswe293T cells infected by the SV2A-silencing virus (Figures 2A,B). The results of immunofluorescence analysis of APP and SV2A (Figure 2C), ELISA statistics analysis of the Aβ (Figure 2D), and brain observation by PET in SV2A-KO mice (Figures 2E,F) also validated the increase of Aβ in a SV2A deficiency situation. It has been demonstrated that SV2A deficiency impairs its interaction with synaptotagmin 1, causing a specific disruption of synaptic GABA release, which in turn down-regulates GABA_*B*_ receptor expression (Ohno and Tokudome, 2017). Therefore, we speculated that downregulation of SV2A may impair the inhibition of APP proteolysis to Aβ via reducing the expression of the GABA_*B*_ receptor, thus leading to the upregulation of APP and Aβ (Dinamarca et al., 2019). Yet there is also evidence showing that in AD, the SV2A loss takes precedence in glutamatergic rather than GABAergic nerve terminals (Govindpani et al., 2017), and the specific regulatory mechanism of SV2A on APP remains to be elucidated.

In the present study, we found that SV2A overexpression down-regulates BACE1 and APOE at both the mRNA and protein expression levels (Figure 3). Studies have shown that the level of BACE1 in the brain may be affected by APOE before the onset of AD (Decourt et al., 2013; Liu et al., 2013; Dai et al., 2018). In addition, APOE regulates multiple brain pathways to varying degrees including lipid transport, synaptic integrity and plasticity, and cerebrovascular function (Yamazaki et al., 2019). SV2A regulates the release of action potential-dependent neurotransmitters, and SV2A dysfunction impairs the release of synaptic GABA and glutamate (Tokudome et al., 2016; Lepannetier et al., 2018). Inhibition of BACE1 can promote the activities of various cell receptor proteins in presynaptic or postsynaptic glutamatergic and GABAergic synaptic membranes (Yan et al., 2016). Therefore, we can speculate that SV2A can further influence AD by mediating BACE1 and APOE to regulate the synaptic receptor. However, some studies have shown that SV2A KO prevents ApoE4 from promoting BACE1 processing of APP, which plays a role in WT cells by promoting the colocalization of BACE1 and APP *in vivo* (Rhinn et al., 2013; Swarup and Geschwind, 2013; Yan and Vassar, 2014; Zhang et al., 2015).

It has also been reported that SV2A density was inversely correlated with tau phosphorylation (Metaxas et al., 2019). Therefore, we investigated the relationship between SV2A and Tau and found that SV2A overexpression in APPswe293T cells resulted in significant decreases in tau mRNA and protein levels (Figures 4A,B). Although P-Tau-S356 and P-Tau-Y322 did not change significantly with SV2A overexpression, a slight upward trend was found in P-Tau-S356 of shSV2A (Figure 4C). It is known that tau cross-links synaptic vesicles, thereby slowing their mobilization and ultimately reducing synaptic transmission during intense stimulation (Zhou et al., 2017). Further, overexpression of tau is found to potently inhibit axonal transport (Chee et al., 2006). Since SV2A is involved in the regulation of synaptic vesicle transport, exocytosis, and neurotransmitter release (Mendoza-Torreblanca et al., 2013), overexpression of SV2A may alleviate AD-related symptoms by reducing the content of Tau and thereby inhibiting the impairment of synaptic vesicle information transmission.

In this study, we found that the involvement of SV2A in the pathogenesis and development of AD appears to be mediated by the PI3K signaling pathway, as upregulation of SV2A down-regulated the expression of PI3K (Figure 5A) and treatment with the PI3K inhibitor LY294002 blocked this effect (Figure 5D). Decreased levels of PI3K subunits, as well as blunted AKT kinase phosphorylation, have been observed in the brain of AD patients (Gabbouj et al., 2019). Insulin-like growth factor-2 (IGF-2) was reported to attenuate memory decline and amyloid plaque formation in AD mouse model by activating the PI3K/AKT/CREB signaling pathway (Xia et al., 2019), and insulin has been observed to promote neuron growth and synapse formation through the PI3K signaling pathway (Liem et al., 2017; Gabbouj et al., 2019). Therefore, we speculate that SV2A may play a role in promoting insulin growth factor secretion. However, further studies are needed to clarify the link between SV2A, the PI3K signaling pathway, and AD pathogenesis and progression.

## Conclusion

In conclusion, our datas indicate that that upregulation of SV2A decreases the relative expression level of AD-related genes. We found that Aβ expressions were significantly increased in certain brain regions of SV2A-KO mice by the PET imaging technique. Moreover, we found that SV2A regulation of the occurrence and development of AD appeared to be mediated by the PI3K signaling pathway. This study provides guidelines and information regarding the influence of the SV2A mechanism on the regulation of AD and possible future research of neurological diseases.

## Data Availability Statement

The raw data supporting the conclusions of this article will be made available by the authors, without undue reservation.

## Ethics Statement

The studies involving human participants were reviewed and approved by Ethics Committee of Huashan Hospital Affiliated to Fudan University. The patients/participants provided their written informed consent to participate in this study. Written informed consent was obtained from the individual(s) for the publication of any potentially identifiable images or data included in this article.

## Author Contributions

YK, JW, RN, CZ, BS, and YG designed the experiments. YK, LH, and WL conducted most of the experiments, with assistance from XL, YZ, and CL. YK, LH, SZ, and XL collected data and contributed to the statistical analysis. FX, ZZ, DJ, and WZ radiosynthesized PET tracer. YK and JW analyzed the data and wrote the manuscript. YK and YG obtained funding. YK and RN revised the manuscript. All authors read and approved the final manuscript.

## Conflict of Interest

The authors declare that the research was conducted in the absence of any commercial or financial relationships that could be construed as a potential conflict of interest.

## Footnotes

1 https://aging.brain-map.org/

## Supplementary Material

Supplementary Figure 1 The SV2A-regulating virus infected into APPswe293T cells was used to generate SV2A-overexpressing and SV2A-silencing cells. **(A)** qPCR detection of SV2A at the mRNA expression level in APPswe293T cells from the following four groups: control, cells infected with the SV2A-overexpressing virus (SV2A), shControl, and cells infected with the SV2A-silencing virus (shSV2A). The expression level was normalized to the mean expression level of the control group. **(B)** The agarose gel electrophoresis pattern of the qPCR product. Gapdh is used as an internal reference. Data are expressed as mean ± SEM. One-way analysis of variance (ANOVA). ^∗^*p* < 0.05, ^∗∗∗^*p* < 0.001. **(C, D)** The construction of a mouse SV2A cKO model. **(C)** Scheme showing the targeting strategy for disruption of the SV2A gene. Exon 3 was selected as the cKO region. **(D)** Agarose gel electrophoresis was used to identify the genotype of the SV2A mouse model. The lanes from left to right are the marker, DNA of the WT mouse, and DNA of the SV2A ± mouse. Data are expressed as mean ± SEM. One-way analysis of variance (ANOVA). ^∗^*p* < 0.05.
