# Supplementary material for: The Synaptic Vesicle Protein 2A Interacts With Key Pathogenic Factors in Alzheimer’s Disease: Implications for Treatment
Source: Front Cell Dev Biol. 2021 Jul 1;9:609908. doi: 10.3389/fcell.2021.609908 (PMC8282058; doi:10.3389/fcell.2021.609908)

Supplementary material

Figure legends

Supplementary Figure S1. The SV2A regulating virus infected into APPswe293T cells was used to generate SV2A overexpressing and silencing cells. (A) qPCR detection of SV2A at mRNA expression level in APPswe293T cells from the following four groups: Control, cells infected with SV2A overexpressing virus (SV2A), shControl, and cells infected by SV2A silencing virus(shSV2A). The expression level was normalized to the mean expression level of Control group. (B) Agarose gel electrophoresis pattern of qPCR product. GAPDH is used as an internal reference. Data are expressed as mean ± SEM. One-way analysis of variance (ANOVA). *p < 0.05, ***p < 0.001.

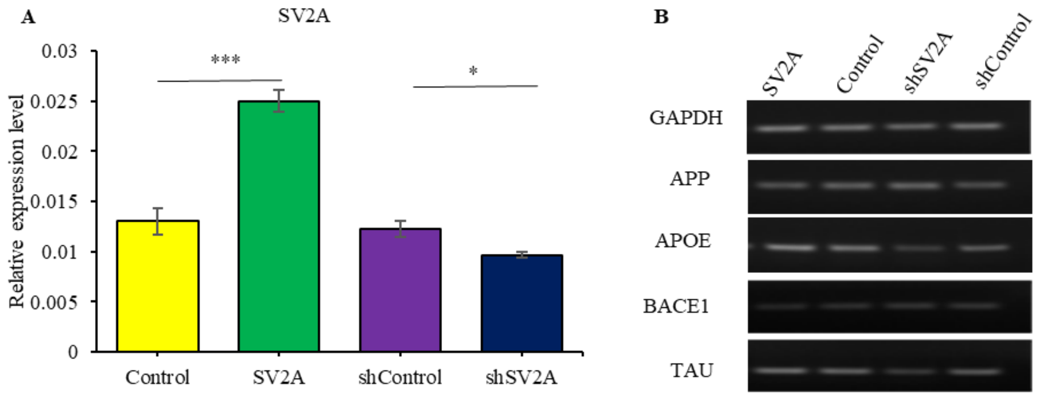

Supplement: Supplementary file 1 [file Data_Sheet_1.PDF]
