# Supplementary figures and images for: The Synaptic Vesicle Protein 2A Interacts With Key Pathogenic Factors in Alzheimer’s Disease: Implications for Treatment
Source: Front Cell Dev Biol. 2021 Jul 1;9:609908. doi: 10.3389/fcell.2021.609908 (PMC8282058; doi:10.3389/fcell.2021.609908)

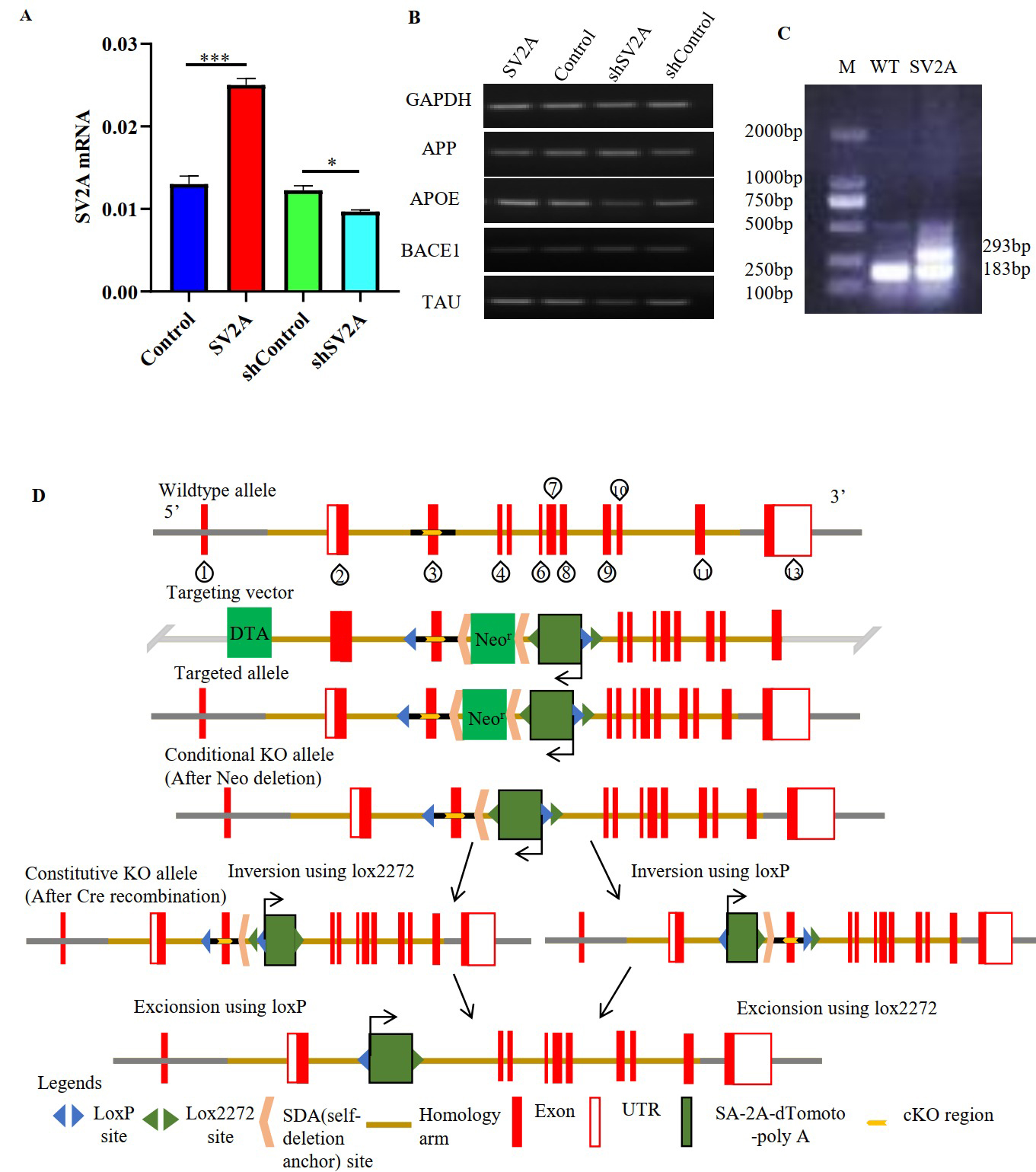

Supplement: Supplementary Figure 1 — The SV2A-regulating virus infected into APPswe293T cells was used to generate SV2A-overexpressing and SV2A-silencing cells. (A) qPCR detection of SV2A at the mRNA expression level in APPswe293T cells from the following four groups: control, cells infected with the SV2A-overexpressing virus (SV2A), shControl, and cells infected with the SV2A-silencing virus (shSV2A). The expression level was normalized to the mean expression level of the control group. (B) The agarose gel electrophoresis pattern of the qPCR product. Gapdh is used as an internal reference. Data are expressed as mean ± SEM. One-way analysis of variance (ANOVA). ∗p < 0.05, ∗∗∗p < 0.001. (C, D) The construction of a mouse SV2A cKO model. (C) Scheme showing the targeting strategy for disruption of the SV2A gene. Exon 3 was selected as the cKO region. (D) Agarose gel electrophoresis was used to identify the genotype of the SV2A mouse model. The lanes from left to right are the marker, DNA of the WT mouse, and DNA of the SV2A ± mouse. Data are expressed as mean ± SEM. One-way analysis of variance (ANOVA). ∗p < 0.05. [file Image_1.jpeg]
